# Alternative Lengthening of Telomeres in Yeast: Old Questions and New Approaches

**DOI:** 10.3390/biom14010113

**Published:** 2024-01-16

**Authors:** Kendra Musmaker, Jacob Wells, Meng-Chia Tsai, Josep M. Comeron, Anna Malkova

**Affiliations:** 1 Department of Biology, University of Iowa, Iowa City, IA 52242, USAjacob-wells@uiowa.edu (J.W.);; 2 Interdisciplinary Graduate Program in Genetics, University of Iowa, Iowa City, IA 52242, USA

**Keywords:** alternative lengthening of telomeres (ALT), yeast, ALT frequency, break-induced replication (BIR), Oxford nanopore sequencing technology (ONT), ALT intermediates

## Abstract

Alternative lengthening of telomeres (ALT) is a homologous recombination-based pathway utilized by 10–15% of cancer cells that allows cells to maintain their telomeres in the absence of telomerase. This pathway was originally discovered in the yeast *Saccharomyces cerevisiae* and, for decades, yeast has served as a robust model to study ALT. Using yeast as a model, two types of ALT (*RAD51*-dependent and *RAD51*-independent) have been described. Studies in yeast have provided the phenotypic characterization of ALT survivors, descriptions of the proteins involved, and implicated break-induced replication (BIR) as the mechanism responsible for ALT. Nevertheless, many questions have remained, and answering them has required the development of new quantitative methods. In this review we discuss the historic aspects of the ALT investigation in yeast as well as new approaches to investigating ALT, including ultra-long sequencing, computational modeling, and the use of population genetics. We discuss how employing new methods contributes to our current understanding of the ALT mechanism and how they may expand our understanding of ALT in the future.

## 1. Introduction

In eukaryotes, the ends of linear chromosomes are protected from degradation by repetitive sequences of DNA known as telomeres that are bound by telomere-specific proteins. These proteins cap the end of the chromosome thereby preventing the chromosome ends from appearing as one-ended double-strand DNA breaks (DSBs) that would trigger a DNA damage response (DDR) (reviewed in [1,2,3]). Maintaining telomeres becomes challenging during replication as telomeric repeats shorten with each round of replication due to the nature of lagging strand synthesis (i.e., the end replication problem; reviewed in [2,4]). To combat this gradual shortening, telomeres are lengthened by the enzyme telomerase. However, many organisms, including mammals, inactivate telomerase in a majority of cells after embryonic development. As a result, telomeres shorten continuously over the cell’s lifespan until the telomeric repeats reach a critically short length. At this point telomeres are no longer able to protect chromosome ends from detection by DDR machinery and start to resemble a one-ended DSB. The resulting DDR leads to cellular senescence in a majority of cells (reviewed in [2,5,6]). In rare cases, however, cells are able to bypass senescence either by reactivating telomerase or by a telomerase-independent recombination-based mechanism employed by 10–15% of cancers known as alternative lengthening of telomeres (ALT) (reviewed in [7,8]). The yeast *Saccharomyces cerevisiae* has proven to be a very useful model to investigate the mechanism of ALT. In yeast, the chromosome ends contain a 250–300 base pair array of heterogenous telomeric repeats typically abbreviated C_1-3_A/ TG_1-3_. These repeats are maintained by telomerase, which is constitutively active in *S. cerevisiae*, but can be deleted or inactivated, thus allowing for the detailed study of the consequences of telomere shortening.

## 2. A Two-Pathway Model of ALT in Yeast: A Historical Perspective

The first description of ALT in yeast came from Lundblad, Szostak, and Blackburn [9,10], by demonstrating that some cells deficient in the essential catalytic component of telomerase, *EST1*, could bypass cellular senescence. More specifically, *est1Δ* cells were obtained by sporulation of *EST1/est1Δ* diploids and passaged for multiple generations. Although the growth rate of these cells steadily declined and the majority reached senescence when telomere repeats had shortened significantly, a subset of cells resumed growth and maintained divisions for more than 100 generations. The authors proposed that as no active telomerase was available, the cells that bypassed senescence (referred to henceforth as “survivors”) must have done so through a telomerase-independent pathway. Indeed, they observed that the formation of survivors was dependent on the Rad52 recombination protein [9]. Because Rad52 is required for all types of recombination in yeast, this observation demonstrated that the formation of survivors in telomerase-deficient cells required homologous recombination (HR). Later studies established that similar to yeast, ALT in humans also depends on recombination [11,12], suggesting the ALT in yeast and humans is similar, making yeast an attractive model to study ALT.

Analysis of ALT survivors in yeast demonstrated that they come in two distinct types that differ in chromosome end structure [9,13,14]. Specifically, Southern blot analysis demonstrated that Type I survivors stabilized the chromosome ends by expanding the number of the Y’ elements (sub-telomeric repetitive elements found at some, but not all, chromosome ends), with some survivors undergoing a 200-fold amplification of Y’ elements and forming long Y’ tandems. Importantly, telomeres (here defined as the heterogenous array of C_1-3_A/TG_1-3_ repeats found at chromosome ends in *S. cerevisiae*) in Type I survivors remained short, resulting in these survivors being highly unstable and therefore growing slowly and at an inconsistent rate [9]. Type II survivors, by contrast, stabilized by lengthening the telomeric repeats themselves up to a length of 12 kb and had only very modest changes to the Y’ elements [9,13,14]. Type II survivors grew at a stable rate comparable to a telomerase-positive culture and maintained this rate even as telomeres continued to undergo periods of shortening and lengthening.

When survivors were obtained by passaging yeast on plates, the majority of survivors were Type I. However, when cells were passaged in liquid culture, Type II survivors were predominant. Moreover, it was observed that when telomerase-deficient cells were transferred in liquid cultures, Type I survivors were initially found in a culture, but at later timepoints the cultures consisted primarily of Type II survivors [15]. How this transition occurred was unclear. One possibility was that Type II survivors formed later than Type I, but easily outcompeted Type I due to the faster and more consistent growth rate of Type II survivors [13,14,15].

**Figure 1:**
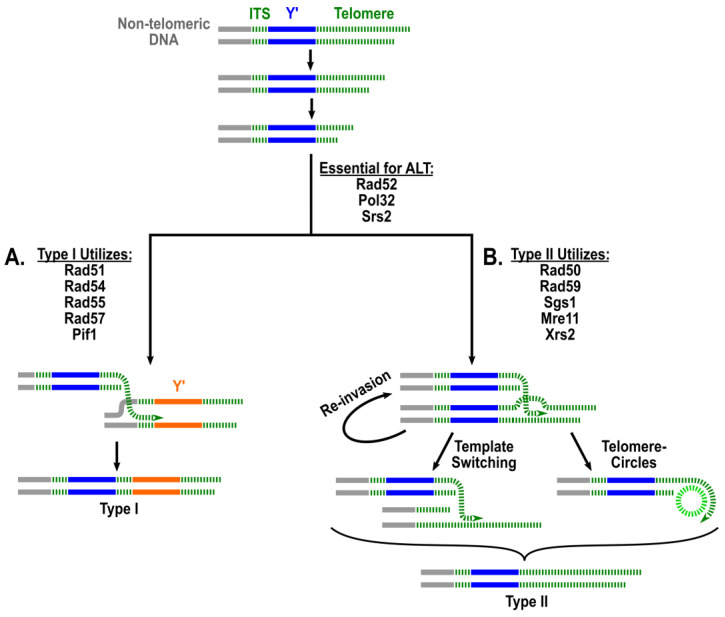
The two-pathway model of ALT in yeast. Following inactivation of telomerase, the telomeres erode until formation of a short end that can be stabilized by one of two pathways: (**A**) a Rad51-dependent pathway leading to the formation of Y’ tandems with short telomeres at the end; (**B**) a Rad51-independent telomere extension that depends on Rad59, MRX, and Sgs1 and stabilizes chromosomes by forming extra-long telomeres. Long telomeres may be formed via annealing/invasion at position of microhomologies followed by template switching, multiple reinvasions, or copying telomere circles. Both pathways depend on Pol32 and Rad52. ITS = interstitial telomere; Y’ = sub-telomeric elements. Green color: telomeres and ITS; blue and orange: Y’ elements.

While both survivor types were eliminated after deleting *RAD52* [9,14], deletions of other known HR genes showed differential effects on distinct ALT types. For example, deletion of *RAD51* made senescence in telomerase-deficient strains more rapid and allowed the formation of only Type II survivors [13,16] as was also the case with the deletions of *RAD54* and *RAD57*, members of the *RAD51* epistasis group [16,17]. This led to the suggestion that Rad51 is required for the formation of Type I survivors. By contrast, the deletion of *RAD50* delayed survivor formation and yielded only Type I survivors [13,16,17]. A similar effect was observed when other members of the MRX (Mre11-Rad50-Xrs2) complex were absent. The MRX complex (analogous to mammalian MRN (Mre11-Rad50-Nbs1)) plays multiple roles in DNA repair, including facilitation of short range DSB resection, DSB end tethering, checkpoint activation and promoting non-homologous end joining. The formation of only Type I survivors in the absence of each of MRX proteins led to conclusion that the MRX complex is specifically required for the formation of Type II survivors. Similarly, the absence of Rad59 yielded only Type I [16], placing *RAD59* in a category of genes specifically required for Type II ALT. Importantly, when *RAD50* and *RAD51* together or *RAD51* and *RAD59* together were deleted [16,17], survivor formation was completely abrogated, similar to *rad52Δ*. Collectively, these observations suggested that ALT occurred via two independent pathways: Type I, which was *RAD51*-dependent (but independent of *RAD50* and *RAD59*), and Type II, which was dependent on *RAD50* and *RAD59* (but independent of *RAD51*) (Figure 1).

The genetic dependencies of these two distinct pathways made ALT similar to one particular type of homologous recombination called break-induced replication (BIR). BIR repairs one-ended DSBs and is known to proceed via two separate pathways—one dependent on *RAD51*, and the other dependent on *RAD50* and *RAD59* but independent of *RAD51* [18,19,20,21]. Thus, Type I ALT survivors were proposed to be formed via *RAD51*-dependent BIR, while Type II survivors via *RAD51*-independent BIR [17]. Crucial evidence that ALT occurs via BIR came from the observation that in the absence of Pol32, an essential BIR factor, both ALT types are completely abrogated [22].

In addition to the genes already mentioned, a variety of other genes were also found to have roles in the ALT pathways. The distinct set of genes involved in the Type I, *RAD51*-dependent pathway of ALT, include *PIF1*, *RIF1*, *RIF2*, and *INO80* complex [13,23,24]. The loss of the helicase *PIF1* or both of the end protection factors *RIF1* and *RIF2* resulted in a complete loss of Type I survivors. The deletion of *RIF1*, *RIF2*, or components of the *INO80* complex individually increased the proportion of Type II survivors obtained from solid media, conditions that favor Type I survivors. Finally, the screen performed by [24] identified several additional genes specifically involved in Type I, including chromatin remodeling genes (*SAP30* and *IES3*) and transcription factors (*RPA14*, *RPB9*, and *SOH1*).

At the same time, a distinct set of genes was identified to be involved in the *RAD50*-dependent Type II pathway. The *SGS1* helicase was required for the formation of Type II survivors [25,26], while the deletion of *EXO1*), or temporary inactivation of polymerase alpha all resulted in an increase of Type I survivors in liquid culture [24,27,28] where Type II survivors are normally predominant. The deletion of components of KEOPS (kinase, endopeptidase and other proteins of small size), a complex consisting of Cgi121, Bud32, Kae1, Pcc1 and Gon7 with roles in tRNA modification, translation (reviewed in [29]), resection during HR [30], telomere uncapping, and telomere length regulation [31], also resulted in an increase in the proportion of Type I survivors. Additionally, the protein ubiquitination genes, *RAD6* and *SLX8*, and the rRNA processing genes, *XRN1* and *RRP17*, were found to have roles in Type II ALT [24].

Finally, some genetic defects affected ALT without a clear impact on one type or the other. For example, the temporary inactivation of polymerase delta affected both survivor types as few survivors formed [27], and those that did form lacked the Y’ amplification characteristic of Type I and had telomeres shorter than expected for Type II. Finally, a deletion of the mismatch repair gene *MSH2* was found to increase the speed of survivor emergence suggesting mismatch repair may inhibit ALT [32]. Overall, the proportion of Type I vs. Type II survivors formed in the absence of a gene became the main criteria for determining the involvement of a gene in one of the ALT types. However, this method was qualitative rather than quantitative and did not allow the effects of individual mutations on the frequency of ALT survivor formation to be determined. Until recently, determining the frequency of ALT in yeast was deemed not to be possible due to the stochastic nature of ALT [1], limiting progress in the understanding of the mechanism of ALT.

## 3. Investigation of the Process of ALT Establishment

In addition to understanding the genetic requirements of ALT, it was important to understand the steps that lead up to the formation of each type of survivor. In the case of Type II survivor formation, survivors gain very long (up to 12 kb) telomeres that contrast with initial lengths of a few hundred bp [9,14]. This observation suggested that Type II telomeres could lengthen gradually as a result of multiple recombination events. To test this, the Zakian lab followed a population of telomerase-deficient cells to senescence and monitored bulk telomere length by Southern blot analysis [14]. They observed that in a telomerase-deficient culture where all telomeres were originally shortened, a subset of cells then rapidly lengthened their telomeres. This observation argued against gradual lengthening and, instead, the authors proposed the formation of telomere circles that are used as a template for telomere extension in a single step. This was consistent with the observations made in several other species. For example, telomere circles have been reported in another type of yeast, *K. lactis*, where all ALT proceeds by a process similar to Type II ALT [33,34,35,36]. The presence of telomere circles is also critical for ALT in mammals (reviewed in [8]). More recently, telomere circles were detected in *S. cerevisiae* ALT survivors by 2D gel electrophoresis [37,38]. However, as telomere circles were not observed prior to survivor formation, it is not known whether they are merely a byproduct of ALT or a template for recombination. Several observations support the idea that the circles indeed contribute to Type II telomere extension. First, multiple labs reported the incorporation of exogenous circles into telomeric regions [39,40], a process that could explain telomere extension. Further, it was observed that telomere circles, as well as telomere ends involved in recombination, co-localized with the nuclear pore complex (NPC), and the loss of NPC components delayed the formation of Type II survivors [37]. Together, these observations suggested that telomeric extension in Type II ALT survivors may occur in a single step via the copying of telomeric circles taking place at the NPC.

Another insight into the formation of Type II survivors was gained by sequencing one chromosomal end (the end of chromosome VI-R) at different steps of ALT establishment [41]. The authors observed that in the early steps, shorter chromosome ends preferentially gained telomere repeats while longer telomere ends were extended later in the process, at the time when survivors started to emerge. Fu et al. [42] on the other hand demonstrated the preferential extension of the shortest ends both before and after the Type II survivor formation. In their work, the short ends were generated by an HO endonuclease, which allowed the authors to analyze telomere extension even in already established survivors. To reconcile the findings from two labs, the authors speculated that short and long telomeres undergo changes in the level of their protection by telomere-binding proteins at different steps of survivor formation, and this can explain why short and long telomeres could be preferentially extended at different steps.

The details of Type I survivor formation were also studied. For example, utilizing a system in which one telomere end (chromosome VII-L) was abruptly shortened by a galactose-induced Cre-Lox-mediated deletion, concomitant with telomerase inactivation, allowed following the acquisition of Y’, a crucial step of Type I ALT survivor formation [43]. The authors found that the acquisition of a Y’ required telomere shortening to approximately 60 bp. Interestingly, the acquisition of the first Y’ required *RAD52* and *POL32* but showed only a modest dependency on *RAD51*, a well-known Type I factor. Instead, it showed a strong dependence on *RAD59* and *SGS1*, genes that were believed to be required only for the formation of Type II. This suggested a more complex interplay between Type I and Type II factors. Specifically, the authors proposed that the formation of Type I survivors consists of two distinct steps: acquisition of the first Y’ in a *RAD51*-independent manner, and subsequent amplification of the Y’ elements in a *RAD51*-dependent way.

Despite the impressive accumulation of knowledge, many details about ALT establishment remain unclear. ALT survivors arise infrequently and stochastically, despite the genetic uniformity of all cells within the culture and the similarity of initial events associated with telomere erosion in these cells. The key is to identify the specific factors and processes that enable rare cells undergoing telomere erosion and senescence to survive via ALT.

To this end, a significant amount of data have been collected regarding the specific molecular events occurring in individual telomerase-deficient cells progressing towards senescence [3,44,45,46,47]. The events occur in the following order: First, telomere erosion leads to the formation of one critically short telomere, behaving similarly to DSBs, becoming subject to DSB resection. Second, DSB resection leads to the accumulation of single-strand (ss) DNA in sub-telomeric regions, inducing DNA damage checkpoint signaling and cell cycle arrest mediated by Ddc2, Rad24, Rad9, Rad53, and Mec1 checkpoint proteins [43,44,45,46,47,48,49,50]. The accumulation of ssDNA also promotes homologous recombination-mediated DNA repair. Using microfluidics, the Teixeira lab traced individual lineages of cells following telomerase inactivation [45,51] and found two cell types: type A, where cells divided constantly before entering senescence, and type B, where cells experienced transient arrest and growth resumption on their way to senescence. Importantly, *POL32*—an essential BIR and ALT gene—was required for type B, thus uncovering a potential link between telomerase-inactive type B and ALT cells. Finally, Coutelier et al. demonstrated that in some cases, arrested cells can resume proliferation without repair, following a pathway called adaptation, resulting in high genomic instability [45]. Irrespectively though, the vast majority of the cells end up permanently senescent.

What remains to be understood are the events in those rare cells that become survivors and resume growth. It has been proposed that these cells upregulate or alter recombination, leading to significantly elongated telomeres, but the mechanisms behind this process remain unclear. Moreover, the transition to survivor formation takes a considerable amount of time, and the bottlenecks of this process remain unknown. The bottlenecks for Type I survivor formation could potentially involve acquiring Y’ elements by chromosome ends lacking them or forming the initial Y’ tandem. Meanwhile, the formation of telomere circles could represent a limiting step in Type II survivor formation. Testing these hypotheses requires tracking individual chromosome ends in both individual cells and populations.

## 4. Determining the Frequency and the Precise Structure of ALT Survivors

**Figure 2:**
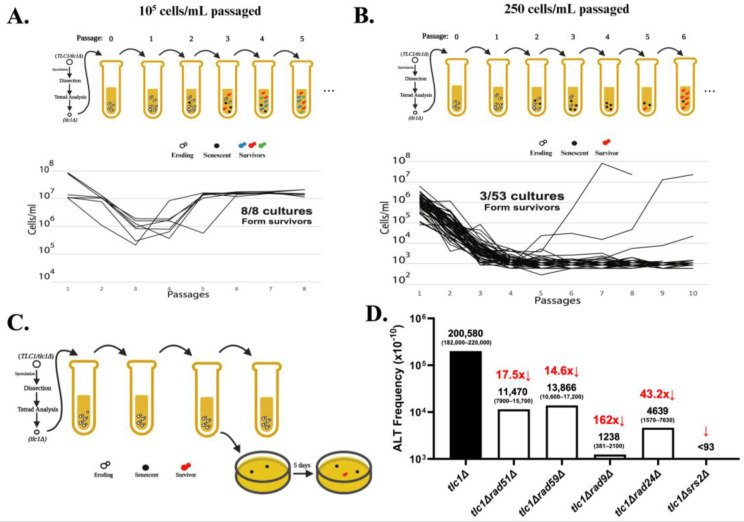
Determining ALT frequency brings new insight into genetic control of ALT (from data in [52] published under license # 5686730647276 from the publisher Elsevier.) (**A**) *tlc1Δ* cells obtained by tetrad dissection were passaged at a high concentration (10^5^ cells/mL) in liquid YEPD for 5 days. ALT survivors, indicated by an increase in growth rate, formed in every *tlc1∆* culture (8/8 cultures) when large populations (10^5^ cells/mL) were passaged. (**B**) *tlc1Δ* cells obtained by tetrad dissection were passaged at a low concentration (250 cells/mL) in liquid YEPD for 5 days. ALT survivors formed only in a small subset of cultures (3/53 cultures) when small populations were passaged, allowing for the use of Poisson statistics for the ALT frequency calculation. (**C**) Modified passaging scheme with plating of cells after second passage; *tlc1Δ* cells were obtained by tetrad dissection and passaged at 250 cell/mL for 3 days. Cells were plated on solid YEPD after day 2 and survivors (colonies on solid media) were counted 5 days after plating. The frequency was calculated as the number of survivors formed divided by cells plated. (**D**) Effect of deletions of recombination and checkpoint genes on the frequency of ALT in *tlc1∆* cells. Medians of ALT frequencies and 95% confidence intervals (in parentheses) are shown. Non-overlapping 95% confidence intervals indicate a significant difference in frequency. The arrows and fold change on the top represent reduction (or increase) of ALT frequency compared with *tlc1∆.* Note: *tlc1*∆ frequency >> *rad59∆ tlc1∆* + *rad51∆ tlc1∆*.

Although analysis of survivor types provided much insight into the genetic requirements of ALT and its mechanism, the absence of known frequencies for ALT under different genetic conditions constrained progress. Recently, Kockler et al. (2021) [52] proposed that a limitation in previous experiments was the use of the serial passaging of cells in liquid culture at high initial density (~10^5^ cells/mL), which produced at least one (and often more than one) ALT survivor in every experiment, thereby precluding frequency calculations (Figure 2A). To address this obstacle, ref. [52] established experimental conditions under which serial passaging of telomerase-deficient *tlc1Δ* mutants (lacking the RNA subunit of telomerase) at a much lower initial cell density (250 cell/mL) (Figure 2B) resulted in only a small fraction of cultures forming ALT survivors. This ensured that each survivor developed from a single event and allowed the authors to calculate the frequency of ALT using a Poisson distribution [52]. They subsequently developed a simplified protocol that involved passaging yeast in liquid only until the cells entered senescence, followed by plating these cultures and monitoring the formation of ALT survivor colonies (Figure 2C). Using this approach, the authors determined the overall ALT frequency to be 2 × 10^−5^ (Figure 2D).

**Figure 3:**
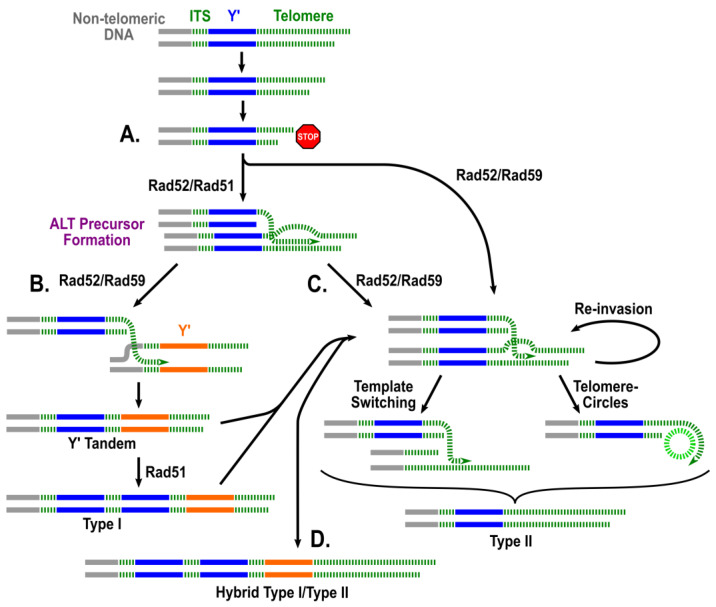
The model of the unified ALT pathway. (**A**) Following the inactivation of telomerase, the telomeres erode until the formation of a short end promotes cell cycle arrest (indicated by the stop sign). Rad51- and Rad52-dependent HR, leading to telomere elongation, forms ALT precursors. The Rad52/Rad59 arrow indicates a Rad51-independent and Rad52- and Rad59-dependent pathways leading to the formation of Type II survivors. (**B**) Rad59 and Rad52 mediate recombination between eroded telomeres and interstitial telomeres sequences (ITS) to form tandem Y’s that are propagated to other chromosomal ends through Rad51-dependent recombination. (**C**) Rad59 and Rad52 mediate recombination at microhomologies between telomeres to form Type II ALT survivors containing long telomeres. Ultra-long telomeres may be achieved via reinvasion (or re-annealing), template switching, or the copying of telomere circles. (**D**) Rad59 and Rad52 mediate the maturation of products of (**B**) to form “hybrid” ALT outcomes containing Y’ tandems and long telomeres. The colors are the same as in Figure 1.

Importantly, having a method to quantify ALT frequency made it possible to quantitatively determine the effects of different genetic mutants on ALT, for example *rad51Δ* or *rad59Δ*. This led to the surprising finding that the formation of ALT survivors was reduced 18-fold in the absence of Rad51 and 15-fold in the absence of Rad59 (Figure 2D), making the sum of *rad51Δ tlc1Δ* and *rad59Δ tlc1Δ* frequencies significantly lower than the overall ALT frequency observed in *tlc1Δ*. This finding contradicted the hypothesis of two independent ALT pathways (Figure 1): Rad51-dependent Type I and Rad59-dependent Type II, which predicted that the sum of ALT frequencies in *rad51Δ* and *rad59Δ* should closely approximate the overall ALT frequency. Based on this observation, Kockler et al. postulated that most ALT survivors may result from a “unified” pathway that requires both Rad51 and Rad59 at its various steps (Figure 3). Also, the observed elimination of all ALT survivors following the deletion of *SRS2,* which is required for resolving toxic joint molecules formed by Rad51 mediated strand invasion [53,54], supported the presence of a common *RAD51*-dependent step during all ALT events (Figure 2D). In addition, defective DNA damage checkpoints drastically decreased ALT frequency, supporting idea that successful ALT requires cell cycle arrest, likely to provide time for the repair of eroded telomeres (Figure 2D). Finally, the same method has been used to determine the frequencies of ALT in several other mutants, including *rad55Δ* and *rif1Δ rif2Δ*.

**Figure 4:**
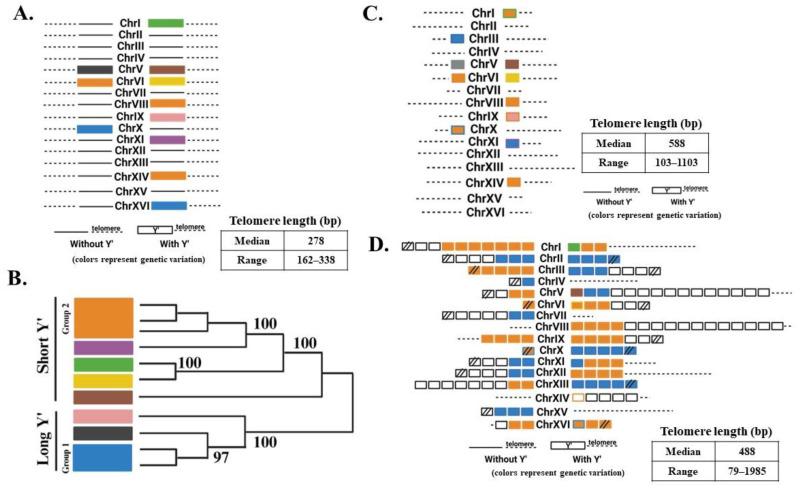
Oxford nanopore sequencing technology (ONT) of ALT survivors supports the “unified” model of ALT (from data in [52] published under license # 5686730647276 from the publisher Elsevier). (**A**) Structure of chromosomal ends in the parental (*TLC1/tlc1∆*) strain determined by ONT. Different Y’ rectangle fill color corresponds to different Y’ sources determined based on (**B**); the strain has 11 Y’ elements with no tandems. Telomere lengths range from 162–338 bp, with mean and median lengths of 275 and 278 bp, respectively. (**B**) Nucleotide distance-based clustering of individual Y’ sequences. Bootstrap values greater than 95 are interpreted as a difference. (**C**) Example of a Type II survivor identified by ONT sequencing. Total number of Y’ is unchanged. 1 lost Y’, 1 gained Y’, 4 swapped Y’, 6 maintained Y’. Telomere length increased compared to the parent, with a range of 103–1102 bp and a median length of 588 bp. (**D**) Example of an ALT survivor where ONT identified the formation of Y’ tandems along with long telomeres, indicating a hybrid type of survivor. The survivor has 152 Y’ elements and telomere lengths ranging from 79–1985 bp with a median length of 488 bp. Different colors represent different Y’ elements. Y’ with undetermined origin, no fill; // indicates that sequencing potentially did not reach the end of the chromosome.

The existence of the “unified” pathway of ALT has been further supported by the detailed characterization of the structure of chromosome ends in ALT survivors. Historically, the structure of ALT chromosomes in yeast has been analyzed by Southern blot to acquire an overall representation of the chromosomal end structure of survivors. A very small subset of ALT survivor chromosomes had been previously sequenced by cloning and Sanger sequencing [41,42,43]; the repetitive nature of telomeres and pre-telomeric regions, however, makes them inadequate substrates for short-read next-generation sequencing (NGS). To determine the precise structure of individual chromosome ends, Kockler et al. employed Oxford nanopore technology (ONT) sequencing to produce ultra-long (exceeding 100 kb) sequencing reads of the parental strain. This approach allowed the detailed characterization of pre-telomeric sequences (11 Y’ pre-telomeric elements, most of them distinguishable from each other by sequence comparison) and telomere length, with an average of 275 bp (Figure 4A,B). ONT sequencing on ALT survivors obtained from *tlc1Δ* mutants identified some structures typical of previously described survivors. For instance, a subpopulation of ALT survivors contained elongated telomeres whereas the number of Y’ elements was close to that in the parental strain, a phenotype typical of Type II survivors (Figure 4C). In some of these cases, Y’ elements underwent losses, gains, and swapping between chromosome ends. Strikingly, ONT analysis also uncovered a subpopulation of ALT survivors that harbored chromosomes with a high number of Y’ elements (a phenotype of classical Type I survivors) and very long telomeres (a feature of classical Type II survivors) on many of the chromosome ends (Figure 4D). Based on the previously unrecognized co-occurrence of tandem Y’ elements and long telomeres (“hybrid” structures), Kockler et al. concluded that the classic ALT types are not mutually exclusive, in support of a “unified” ALT model. Further support for the “unified” model was provided by analyses of chromosome end structures of ALT survivors obtained in various ALT mutants. For example, *rad59Δ tlc1Δ* produced both Type I and Type II survivors, in contrast to the previous conclusion that only Type I could be obtained in this background. Additionally, though *rad51Δ tlc1Δ* produced only Type II survivors, these survivors had multiple changes in the structure of their Y’ elements and resultingly supported an interplay between the elements of Type I and Type II survivor pathways. Together, the occurrence of “hybrid” structures, combined with the frequency results (Figure 2D), supported the “unified” ALT model, which predicts a combination of features of Rad51- and Rad59- pathways within individual survivors (Figure 3).

## 5. Determining the Mechanisms Governing the Development of ALT Survivors

To determine the molecular mechanisms driving the “unified” ALT pathway, Kockler et al. decided to identify the characteristic features of ALT precursors—the population of cells that produce ALT survivors following telomere erosion. Towards this goal, large populations of *tlc1Δ* yeast cells undergoing telomere erosion were analyzed by Southern blotting to identify telomere changes preceding ALT. It was observed that as the telomeres eroded, the distribution of telomere lengths became asymmetric with an excess of short telomeres and a tail of rare long telomeres [52]. The authors proposed that the population of cells driving the skewed distribution represented an ALT precursor population, defined as cells that had arrested their cell cycle to repair by HR at least one critically short telomere. In support of this, the asymmetric distribution was almost fully eliminated in the absence of DNA damage checkpoints (*rad9Δ* or *rad24Δ*) or HR (*rad51Δ* or *rad52Δ*). In addition, HR-deficient *tlc1Δ* strains that had a nearly symmetrical distribution of telomere lengths underwent early senescence (at approximately population division 27 (PD27)), consistent with [9,13,14,16,17,51]; while HR-proficient strains that were characterized by an asymmetric distribution of telomere lengths at the same timepoints were still replicating. Together, these findings suggested that the extension of chromosomal ends by HR leads to a skewed telomere length distribution and delays senescence.

The results of this analysis were further supported by computational modeling, which allowed the authors to determine several important characteristics of telomerase-deficient cells undergoing telomere erosion. For example, the model of telomere erosion in HR-deficient cells allowed the authors to determine the rate of telomere erosion to be ~6 bp per division [52], which was higher than the ~4 bp/division that was previously reported based on a study of telomere erosion in HR-proficient cultures [46,55,56]. The authors hypothesized that the reason they detected a faster erosion rate was that HR was actively occurring in previous studies, which could counteract telomere erosion. To account for this effect of HR, the proposed model was modified such that telomere erosion continues until at least one telomere in a cell was shortened to the critical length, at which stage HR is induced with a donor telomere or pre-telomeric region being used as a template. This computational modeling predicted that activation of HR at telomeres would delay senescence by more than ten population divisions and that a signature of active HR in these populations would be a shift to an asymmetric or skewed distribution of telomere lengths. Together, the results of computational modelling as well as our experimental results supported the idea that the formation of skewed telomere populations reflects the extension of chromosomal ends by HR which may represent the first step in the formation of early precursors of ALT.

The next important step was to determine how these early precursors of ALT are transformed into ALT survivors and to identify the molecular functions involved in the later transitions. Towards this end, [52] took advantage of PacBio sequencing technology of individual telomeres in samples of *tlc1Δ* cells collected at several time points across telomere erosion and demonstrated that *RAD59*, which was not involved in the formation of early ALT intermediates, was needed at a later step of ALT survivor formation. This sequencing effort showed that following a long period of telomere erosion after the cell cultures became fully senescent, a small fraction started transitioning into survivors, at which time PacBio sequencing detected the emergence of long survivor telomeres. Importantly, the transition to longer telomeres, characteristic of ALT survivorship, was defective in the absence of the Rad59 protein, known to be involved in single-strand annealing (SSA). The authors proposed that Rad59 (together with Rad52) mediates recombination (SSA) at microhomologies (consistent with [57,58,59,60]). This is a particularly relevant property to yeast telomeres consisting of degenerative repeats [61], which create ample opportunities for an eroded telomere to find microhomology among other telomeres and, potentially, increase the length of the telomere template significantly. The computational model of telomere erosion and repair showed that adding the possibility of recombination at microhomologies along telomeres could indeed generate survivor-like telomeres. Thus, Rad59 represents the first player that was specifically involved in the later steps of ALT development. Finding other factors/proteins involved in this transition from early precursors to ALT survivors represents an important goal for research in the future.

## 6. Summary and Future Prospects

Since 10–15% of cancer cells utilize ALT for telomere maintenance, understanding ALT’s underlying mechanism is important for potential therapeutics and treatments. Advances in human cell models allow for the study of ALT maintenance, but investigation of ALT establishment in human cells remains difficult. Therefore, baker’s yeast remains a robust model to study the molecular processes and genetic factors associated with the generation of ALT survivors. Recent progress in the development of quantitative methods for the analysis of ALT in *Saccharomyces cerevisiae* as well as technological advances in ultra-long sequencing have created a basis for rapid progress in our understanding of the ALT mechanism in yeast, and some of the important, outstanding, questions that could be answered now are listed below.

1. The newly developed precise methods that allow for the calculation of the frequency of ALT and for the determination of the structure of individual chromosomal ends can be applied to determine the consequences of any genetic, structural, or environmental change on the frequency and telomere structures of ALT survivors. This will allow us to determine the mechanism of ALT through the identification of the specific roles that are played by various genetic factors in ALT.
2. Combining computational modeling with ultra-long (for example, ONT) sequencing will allow for the tracking of structural changes of individual chromosomes during the entire process of ALT: from the beginning of telomere erosion through the formation of ALT survivors and their maintenance. Such analysis is expected to identify the discrete steps of ALT development and to characterize their structural parameters and genetic control. Specifically, it will be very important to establish connections between early ALT precursors that are formed at the beginning of the process and the final ALT survivors. It will be also important to identify the mechanisms and the main components conducive to the formation of ALT precursors including delineating relevant chromosome structures, determining the events that trigger ALT, and identifying the proteins and signaling pathways involved.
3. It has been proposed that extrachromosomal telomeric circles may serve as templates for telomeric extension during the formation of Type II and possibly “hybrid” type survivors. To test this experimentally, telomeric circles could be introduced exogenously (by transformation) into telomerase deficient yeast. We would predict that if indeed extrachromosomal telomeric circles can serve as templates for telomere extension, and not merely represent a byproduct of survivor formation, providing the circles exogenously would (1) increase the frequency of survivor formation; (2) result in the earlier formation of survivors from precursors; (3) result in a greater pro-portion of survivors having a type II or hybrid end structure, and (4) possibly compensate for the absence of some proteins that were proposed to be specifically involved in Type II formation (e.g., Rad59, Sgs1, etc.).
4. We believe that the results of research focused on ALT establishment in yeast will establish a roadmap to guide future studies of ALT in human models. For example, based on recent success in converting ALT-negative into ALT-positive cells (for example, via infection with the KSHV virus [62]), we envision that the methods and approaches developed in yeast will soon be able to be translated into detecting and characterizing the individual steps of ALT establishment in human cells. Specifically, the population genetics approach to determine ALT frequency can be used to test the current hypothesis that several genetically independent ALT pathways operate in humans (e.g., *RAD52*-dependent and -independent ALT pathways [63,64]) or, conversely, whether these pathways intermingle into a more complex unified pathway. Also, future studies of human ALT will greatly benefit from the molecular genetics and genomic approaches to the structural analysis of chromosome ends in ALT precursor cell populations, as well as in ALT outcomes. These approaches hold potential translational significance. They offer the ability to provide insights into specific molecular signatures of pre-senescent cells at different steps during ALT establishment, potentially serving as diagnostic biomarkers for the early detection of ALT-mediated aging/carcinogenic events. Additionally, we expect that the research in yeast will identify the primary proteins driving ALT, which would inform the study of human homologs of these yeast ALT genes and the development of new targeted, mechanism-based, anti-ALT therapeutics.

## Data Availability

Data Sharing is not applicable.
